# A systematic review of health sciences students’ online learning during the COVID-19 pandemic

**DOI:** 10.1186/s12909-022-03579-1

**Published:** 2022-07-03

**Authors:** Abdull Assyaqireen Abdull Mutalib, Abdah Md. Akim, Mohamad Hasif Jaafar

**Affiliations:** 1 Department of Biomedical Sciences, Faculty of Medicine and Health Sciences, Universiti Putra Malaysia, 43400 Serdang, Selangor Malaysia; 2 Academy of Contemporary Islamic Studies, Universiti Teknologi MARA, 72000 Kuala Pilah, Negeri Sembilan Malaysia

**Keywords:** Online learning, COVID-19, Effectiveness, Health Sciences

## Abstract

**Background:**

This study aims to analyse the effectiveness of distance learning during the COVID-19 pandemic among undergraduate health sciences students using systematic review. Online learning has been chosen as the best approach to continue offering education in this pandemic era. Method: The screening process was done using Scopus, ScienceDirect and PubMed based on the eligibility criteria. Out of 1486 studies, 1269 were screened. A total of 64 eligible studies obtained were included in the quantitative analysis. Results were categorized into i) student attitudes (perceptions/satisfactions/engagements), and ii) student learning outcomes, and compared to the Kirkpatrick model.

**Results:**

Although facing difficulties, 50% of the studies was moderately satisfied with distance learning, while 36% was highly satisfied and 17% dissatisfied. Most studies (26%) reported flexibility in online learning. Internet issues (19%) and low interaction between learners and instructors (19%) were the most prevalent problems mentioned. Online education engages students better than traditional learning. The learning outcome was assessed using two categories: i) academic performance and ii) skill development. Most studies (72%) stated that online learning improves academic performance, 14% reported a drop, and 14% stated no effect, while an increase in clinical skills and communication skills were reported. Kirkpatrick evaluation revealed 80% of the studies obtained was evaluated at level 1 (reaction), 8% at level 2 (learning), 12% at level 3 (behaviour) and none at level 4 (results).

**Conclusion:**

Overall, this systematic review found that the online learning performed better than expected during COVID-19, but the data gained is insufficient to say it is beneficial when compared to other types of teaching approaches.

## Background

A significant increase in the usage and acceptance of educational technology was already noticed by researchers in 2019, a year prior to the COVID-19 pandemic [1–4]. The use of suitable information and communications technology (ICT) in education is deemed critical as it can benefit all students [5]. Many researchers suggest that through a better understanding of the obstacles and aspirations of students, higher educational institutions may develop measures to help them continue getting the best education in the event of a pandemic that forces a switch from a traditional mode of learning (physical, face-to-face sessions) to a remote one [6].

One of the defining characteristics of online learning is that students can participate in the learning sessions at any time [7]. Although face-to-face learning remains the preferred way of delivery, the use of a blend of synchronous and asynchronous online learning has grown in popularity in recent times [8]. Different persons and age groups respond to online learning in different ways. Challenges such as download faults, installation issues, login issues, audio and video issues, as well as lack of interaction between students and teachers remain some of the most pressing obstacles to this increasingly popular delivery method. On a similar note, some students believe that pre-recorded videos are the most effective way to conduct lessons during the pandemic [8].

Past studies have suggested that the outcome of distance / online learning is, at large, mixed. A study by Hurlbut (2018) reported that students perform better in physical classes compared to online ones [9]. This is further validated by Sintema [10], who reported that students’ academic performance is significantly affected by their presence in physical classes, as in-person individual activities are essential for students to comprehend the subject matter [11]. Some researchers have also attempted to investigate the impacts of online learning on students’ attitudes. Student engagement, satisfaction, and perceptions are examples of student attitudes that can be observed and determined [12]. As attitudes are subjective, evaluating an individual’s or a group’s attitudes is challenging and numerous factors must be considered in order to properly evaluate them. Observation, direct questions on their views about the subject, performance assessments, and observing the respondents’ reaction on organized stimuli are approaches that can be used to gather data for attitudes [13].

Meanwhile, another group of researchers reported that students recorded better performances in a non-physical learning setting. According to Heitmann et al. (2022), students who received non-bedside teaching performed better than those who attended physical classes [14]. In addition, Hannay and Newvine (2006) found that students that undergo web-based learning performed a lot better than those who received face-to-face education [15]. Some researchers have also discovered that the impact of online learning to students’ performance is either not significant or negative in nature. Kemp & Grieve (2014) stated that no significant difference on test performance was noticed when they compare students studying in physical class to those learning online [16]. Others such as Mukhtar et al. (2022) reported that students’ performance through online learning is expected to deteriorate due to problems with technology and lack of communications with instructors whenever the students face difficulties grasping the learning content. Students also stated that they had difficulty paying attention during lectures. Several instructors have reported misbehaving students during online assessments where these students referred to their lecture notes and searched the internet for solutions during the assessment, despite being told not to [17].

Despite having some evidence that online learning is as successful as conventional methods of learning, there is relatively little research concerning which specific method works (specifically within the domain of Health Sciences) and how online learning improves teaching and learning – especially during the COVID-19 pandemic. Considering the learning styles, pedagogical designs, and students’ expectations unique to the Health Sciences, integrating online learning into Health Science education may be a particularly tricky endeavor. It is for this reason that this systematic review aims to analyze recent publications and research on online learning during COVID-19 pandemic among Health Sciences students to extract valuable key learnings and insights.

## Methodology

### Inclusion criteria

Studies involving undergraduate students from the medical, biomedical, dentistry, nursing and veterinary disciplines who have experienced online learning during the COVID-19 pandemic were chosen for review. The results of interest were learning outcomes (based on academic performance) and attitude of students during COVID-19 online learning (based on satisfaction, perceptions, and engagement). Both quantitative and qualitative studies were included.

### Exclusion criteria

Studies that do not involve undergraduate students (such as those that are focused on postgraduate students, primary school students, and secondary school students) as well as those that investigated non-online learning were excluded. In addition, studies that include online learning but the implementation was not during the COVID-19, those that do not report students’ learning outcomes and students’ attitudes, as well as those not conducted in English were also excluded.

### Search strategy and database used

PubMed, Scopus, and ScienceDirect were used to find articles for review. These databases were shortlisted as they subscribe to many journals that contain published articles related to the Health Sciences. All searches were done between 23^rd^ February 2021 to 23^rd^ June 2021. The Boolean operators (OR & AND) were used to combine various components when constructing the search keywords. Redundant papers were removed.

The search terms used were:

- **PubMed**
  (Online learning OR distance learning) AND (undergraduate student OR university student) AND (learning outcome OR skills OR competences OR satisfaction OR perspective OR reaction OR engagement) AND (COVID-19 OR coronavirus OR COVID19)
- **Scopus**
  (Online learning OR distance learning) AND (undergraduate student OR university student) AND (learning outcome OR skills OR competences OR satisfaction OR perspective OR reaction OR engagement) AND (COVID-19 OR coronavirus OR COVID19)
- **ScienceDirect**
  (Online learning) AND (university student) AND (learning outcome OR skills OR competences OR satisfaction OR perspective OR engagement) AND (COVID-19)

### Screening process

The first screening was conducted after all filtered articles were exported to Mendeley. Articles’ titles were screened and the abstracts of potentially relevant articles were read in full. When screening the abstract, we eliminated all articles that did not meet any of our requirements. Articles that passed the first screening were then subjected to full-text screening. They were read in full, and only those that met all our inclusion requirements were finalized and included in this systematic review. These articles were then subjected to a data extraction and analysis process after the second screening was completed.

### Data analysis

All data gathered was categorized based on the results obtained from a data extraction table. New tables were created for each of the outcomes – including student perceptions, satisfaction, experience, engagement, and learning outcome. A summary of the various outcomes was conducted, which was then compared to the Kirkpatrick Model of evaluation based on four levels – Reaction, Learning, Behavior, and Results.

### Quality assessment

A quality assessment was carried out using the Alberta Heritage Foundation for Medical Research’s checklist (AHFMR) [18]. A two-score system was used to analyze the qualitative and quantitative aspects of the included studies. Quantitative and quantitative studies were examined based on 14 and 10 AHFMR items, respectively.

## Results

**Fig. 1:**
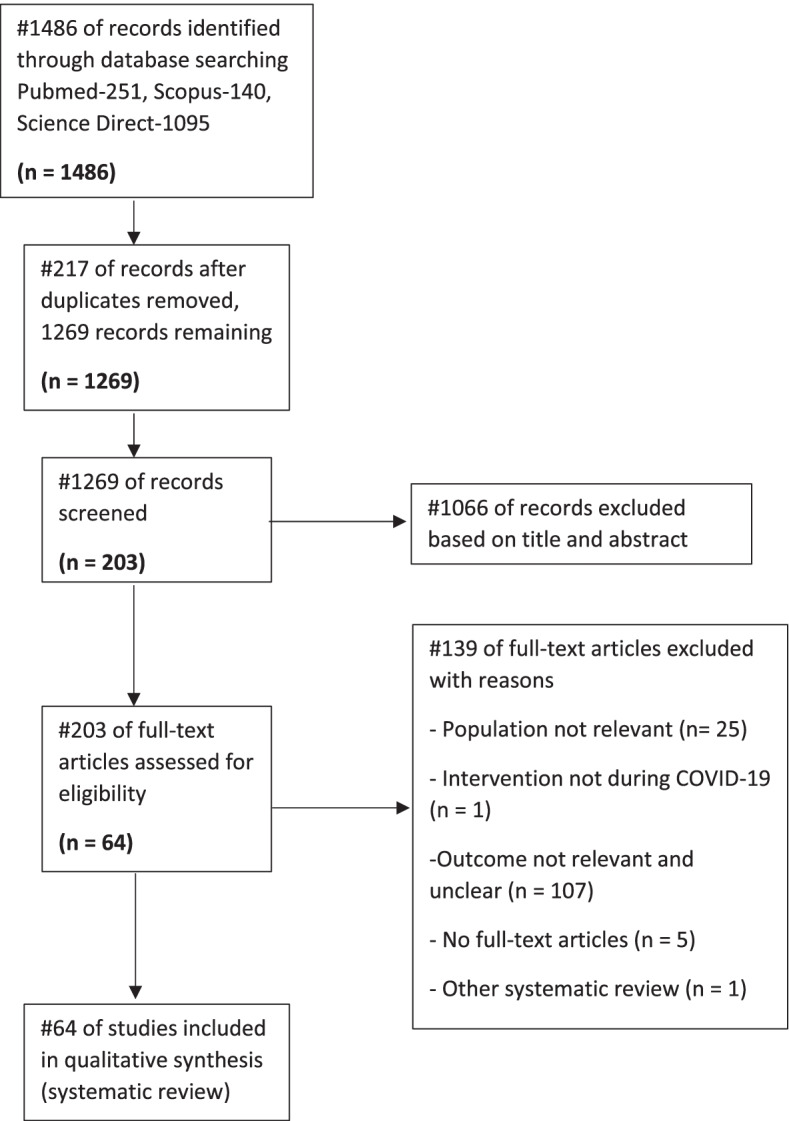
Flow of literature search according to PRISMA guidelines

A total of 1,486 studies were retrieved from three databases: PubMed, Scopus and ScienceDirect (Fig. 1). Two hundred seventeen studies were removed as duplicates by using Mendeley as the management tool and through manual screening of similar titles and abstracts. The remaining 1,269 studies were screened by title and abstract according to the eligibility criteria expounded below. Post-screening, 1,066 studies were excluded for various reasons – such as the population involved are not relevant, the intervention was not during COVID-19, the outcome presented was not relevant and clear, no full-text article, and article including another systematic review – while the remaining 203 studies were further analyzed using full-text assessments. One hundred thirty-nine studies were excluded as they did not meet the described eligibility criteria that include; the studies must only involve undergraduate students from medical and Health Science students from any country who had some experience with online learning during the COVID-19 pandemic, studies must involve online learning applications that are compared to any other teaching methods, as well as studies must include student attitudes and learning outcomes as the results to be assessed. Only 64 studies that meet the above strict criteria were chosen to be included for qualitative synthesis.

### Characteristics of the included studies

Table 1 depicts the characteristics of the 64 filtered studies included in the systematic review. Among them, 56 were cross-sectional studies. Besides that, two papers were qualitative studies [19, 20], two were mixed-method studies [21, 22], one was a retrospective comparative cohort study [23], one a randomized controlled trial [24], one a prospective study [25] and one a case–control study [26]. Most of the papers were published in 2020 (48 of them), while the remaining 16 were published in 2021.

**Table 1:** Characteristics of included studies categorized based on different variables

Variables	Number of studies	Percentage (%)
**Type of studies**		
Cross-sectional study/Descriptive study/Survey	56	87.5
Mixed method study	2	3.13
Qualitative study	2	3.13
A retrospective comparative cohort study	1	1.56
Randomized controlled trial	1	1.56
Prospective study	1	1.56
Case–control study	1	1.56
**Type of population (All undergraduates)**		
Medical students	43	67.69
Health Science students	6	9.23
Dentistry students	5	7.69
Nursing students	4	6.15
Veterinary students	2	3.08
Medical + Nursing students	2	3.08
Medical + Dentistry students	1	1.54
Pharmacy students	1	1.54
**Type of comparison**		
Traditional learning	62	96.88
Blended learning	2	3.13
**Type of outcome**		
Student perceptions	40	43.96
Student satisfaction	36	39.56
Learning outcomes	14	15.38
Student engagement	1	1.10

The population involved in these studies include a mix of undergraduate students from various Health Sciences-related disciplines. Forty three studies involved the participation of undergraduate medical students, six studies involved undergraduate Health Sciences students, five studies involved undergraduate dental students, four studies involved undergraduate nursing students, two studies involved undergraduate veterinary students, two studies featuring a combination of medical and nursing students, one study featuring a combination of undergraduate medical and dentistry students, and one study involved undergraduate pharmacy students.

Most reviewed studies compared online learning applications to traditional learning approaches (62 studies). Meanwhile, there are two studies that compared online learning with blended learning approaches – a combination of online and traditional learning [27, 28]. The outcomes for all studies were categorized into four main categories: learning outcomes, student perceptions, student satisfaction, and student engagement. Based on the results 40 studies reported students’ perceptions, 36 reported students’ satisfaction, 14 reported learning outcomes, and one reported students’ engagement.

### Students’ perceptions on online learning

Students’ perceptions on online learning were assessed using various assessment tools and was compared to their perceptions of traditional learning (Table 2). Most studies used online questionnaires as the preferred assessment tool. One study, however, leveraged online interviews. The study designs include cross-sectional, case–control, mixed, and qualitative study designs. Most studies are at Kirkpatrick level 1, while three of them are at level 2 [29–31] and two are at level 3 [26, 32].

**Table 2:** Summary of the included studies for student perception

Study	Intervention (what used, who involved)	Assessment tool	Comparison	Study design	Findings	Kirkpatrick
Al-Balas et al. (2020) [33]	OL; UG medical students from all medical universities in Jordan	Online questionnaire	Traditional learning	(*N* = 652) Cross-sectional study	55.9% agreed that OL had many advantages, such as interaction between learners and instructors, 62.1% agreed that there was poor interaction between learners and instructors, 48.3% reported low quality of teaching and 69.1% reported internet problem	1
Alqurshi (2020) [34]	OL; UG pharmacy students from institutions in Saudi Arabia	Online questionnaire	Traditional learning	(*N* = 703) Cross-sectional study	More than 20% were having problems with an internet connection, 35% agreed that student-instructor interaction was very limited. 35% agreed that it was harder for them to concentrate in OL, 35% of the students reported having problems gaining knowledge and laboratory skills	1
Alsoufi et al. (2020) [35]	OL; UG medical students from all medical universities in Libyan	Online questionnaire	Traditional learning	(*N* = 3348) Cross-sectional study	54.1% believed that interactive discussion can be achieved via OL. 21.1% agreed that OL could be applied in clinical training, while 54.8% disagreed with the statement, 24% were neutral	1
Amir et al. (2020) [36]	OL; UG dental students from Dentistry faculty in Indonesia	Online questionnaire	Traditional learning	(*N* = 301) Cross-sectional study	52.6% agreed OL provides a more proactive learning method, 87.6% provide more time to study, and 87.3% agreed that OL gives them more time to review their study material.	1
Anwar et al. (2021) [37]	OL; UG medical and dental students from CMH Lahore Medical College	Online questionnaire	Traditional learning	(*N* = 283) Cross-sectional study	38.2% believed that OL provided flexibility. 31.4% believed that OL saved time. 33.2% disagreed that OL improved communication between students and lecturers. 79% of students agreed that they had access to technological equipment, while 83.8% felt that OL is appropriate during pandemic	1
Bączek et al. (2021) [38]	OL; UG medical students from Polish	Online questionnaire	Traditional learning	(*N* = 804) Cross-sectional study	69% like OL due to ability to stay at home, 69% agreed that they had continuous access to OL material and 64% agreed that OL provides the opportunity to self-study. 54% reported having technical problem, and 70% believed that there was less interaction between medical students and patients	1
Chandrasinghe et al. (2020) [39]	OL; UG medical students from Sri Lanka	Online questionnaire	Traditional discussion	(*N* = 1047) Cross-sectional study	87% agreed that they gained many advantages. 83.4% agreed that OL helped in their clinical practices, and 79.3% believed that OL helped them build interest in clinical medicine. -31% complained about having internet problems, 25% having problems joining the OL	1
Co et al. (2021) [26]	OL; UG medical students in Hong Kong university	Online questionnaire	Traditional learning	(*N* = 62) Case–control study	Obstacles—lack of f2f interaction, internet connectivity, and different time zone (overseas students). Benefits includes able to demonstrate the skill easily,	3
Coffey et al. (2020) [40]	OL; UG medical students from the School of Medicine at UC San Diego	Online questionnaire	Traditional learning	(*N* = 132) Cross-sectional study	Most students had good internet connectivity, only 11% reported internet problem. Students reported flexibility in OL and increase engagement. Barrier includesdifficulties in time management, lacking clinical experience and anxiety	1
De Ponti et al. (2020) [41]	OL; UG medical students from the University of Insubria	Online questionnaire	Traditional training	(*N* = 115) Cross-sectional study	77% agreed that OL was very effective in the clinical assessment, 94% agreed it could be used in diagnosis, and 81% agreed that it could be used in treatment management. 28% believed that OL is quite difficult to implement due to technical issues	1
Dost et al. (2020) [42]	OL; UG medical students from 39 medical schools in the United Kingdom	Online questionnaire	Traditional learning	(*N* = 2721) Cross-sectional study	19.82% agreed it could save time, 19.52% agreed it provided flexibility, 14.24% agreed it could save some costs. Disadvantages include family distraction (26.76%), internet connectivity problems (21.53%), anxiety (17.31%) and lack of space (11.03%)	1
Elsalem et al. (2021) [43]	OL; UG students from Jordan University of Science and Technology	Online questionnaire	Traditional learning	(*N* = 730) Cross-sectional study	68.22% preferred f2f exam, 1/3 preferred online exam. 49.86% agreed that more hard work and time were required in preparation for the online exam. 62.33% reported they did not achieve their objectives in their study	1
Jaap et al. (2021) [29]	OL; UG medical students from UK medical school	Online questionnaire	Traditional learning	(*N* = 447) Cross-sectional study	18.5% had problems finding a good environment to sit for their exam, and 84% had very good internet connection. 51.3% reported feeling anxious before the exam because they were afraid about their internet connection issues	2
Guiter et al. (2021) [44]	OL; UG medical students from Weill Cornell Medicine-Qatar	Online questionnaire	Traditional learning	(*N* = 29) Cross-sectional study	Students felt that OL provides easier ability to communicate, which leads them to engage more easily. The environment at home is more relaxing and saves time. Barrier- internet connectivity problems	1
Gupta et al. (2021) [45]	OL; UG medical students from the Delhi-NCR region	Online questionnaire	Traditional learning	(*N* = 248) Cross-sectional study	41.2% agreed that OL could provide flexibility in time and place. Few challenges include 35.9% internet connectivity problem, 29.8% reduce interaction, 27.1% problem with the sound and lack of clinical skills	1
Ibrahim et al. (2021) [46]	OL; UG medical students from King Abdul Aziz University	Online questionnaire	Traditional learning	(*N* = 340) Cross-sectional study	59.7% agreed that OL could replace f2f. 59.2% also feel like OL was less time-consuming than f2f teaching.74.6% agreed that interaction during OL was present between instructors and students, and 54.1% of students agreed that the OL could make them felt motivated. 84.2% agreed OL would affect their clinical skill. 72.1% agreed that their exams could be affected due to internet connectivity problems. 57% agreed that there are limited resources, 32.2% agreed they had technology problem	1
Jiménez-Rodríguez et al. (2020) [47]	OL; UG nursing students from University in Almeria—Spain	Online questionnaire	Traditional practical	(*N* = 93) Cross-sectional study	Students rated 100% in practical utility, 49.4% agreed they had improved their technical skills, and 63.43% agreed that this helps them in their clinical practices	3
Khalil et al. (2020) [19]	OL; UG medical students from Unaizah College of Medicine and Medical Sciences, Saudi Arabia	Online questionnaire	Traditional learning	(*N* = 60) Qualitative study	OL has many benefits, such as saving time, flexibility but in practicing those techniques, some of them also encountered many disadvantages such as internet connectivity, behavioral challenges, and communication problems	1
Khan et al. (2021) [21]	OL; UG medical students from North India	Online questionnaire	Traditional learning	(*N* = 103) Mixed study	Students reported that the OL was very enjoyable, engaging, and motivated them to learn. The disadvantage was lacking practical skill classes, technical issues such as internet connectivity, lack of interaction, and hard to learn how to adapt to the new online stuff	1
Kim et al. (2020) [48]	OL; UG medical students from Seoul National University	Online questionnaire	Traditional learning	(*N* = 456) Cross-sectional study	OL vs offline: 63% vs 29%. 75% preferred recorded video to live online (11.3%)	1
Kumar et al. (2020) [49]	OL; UG medical students from Medical School in Arabian	Online questionnaire	Traditional learning	Cross-sectional study	96% agreed that the online communication was very clear, 85% can maintain the online interactivity, and 92% were interested in continuing with the OL even after the pandemic. 74% of the students agreed that all the software is very user-friendly. few problems mentioned by students, which include limited interaction and clinical training problems	1
Langegård et al. (2021) [22]	OL; UG nursing students from Gothenburg University	Online questionnaire	Traditional learning	(*N* = 132) Cross-sectional study	18% reported having technical difficulties with OL. 2/3 students reported having communication problem,. The students reported OL impacts their motivation in learning. More than 50% reported having problem with their study discipline (hard for them to track everything, especially when they are at home)	1
Mahdy (2020) [50]	OL; UG veterinary students from 92 different countries	Online questionnaire	Traditional learning	(*N* = 1392) Cross-sectional study	Scoring obtained for OL was 5.1 ± 2.4 while for OL practical was 3.6 ± 2.6. Benefits- more convenient and flexible, more time available and save time. Barriers—availability of learning devices, harder to teach in practical, shortness of time and the availability of internet for people who lives in a rural area	1
Martinez et al. (2020) [30]	OL; UG medical students from Florida Atlantic University	Online questionnaire	Traditional learning	(*N* = 47) Cross-sectional study	Students felt that OL was relevant to their medical education. Students reported that these sessions helped them in their exam. They also agreed OL helped them increased interaction even though there were some technical problems	2
Menon et al. (2021) [51]	OL; UG medical students from college hospital in South India	Online questionnaire	Traditional learning	(*N* = 370) Cross-sectional study	The OL barriers reported were connectivity problems, (44.8%) and lack of peer interaction	1
Merson et al. (2020) [52]	OL; UG equine science course	Online questionnaire	Traditional learning	(*N* = 44) Cross-sectional study	Positive feedback on OL, but still, based on Bayesian inference for ANOVA, most of the undergraduates still prefer in-person lessons (*P* < 0.05)	1
Muflih et al. (2021) [53]	OL; UG health science students from Jordanian universities	Online questionnaire	Traditional learning	(*N* = 1210) Cross-sectional study	66.8% agreed that OL made them felt comfortable communicating with their instructors and instructors, 62.6% agreed that the instructor responded quickly to their question. Barriers—75.1% agreed to lack of technical experience, 74.3% lack of experience on online tools, 57.4% lack of motivation, and 62.7% inability to networking	1
Olum et al. (2020)[27[	OL; UG medical and nursing students from Makerere University, Uganda	Online questionnaire	Blended learning	(*N* = 221) Cross-sectional study	Higher interaction (96.3%) in OL. However, 60% believed that they need to train themselves with the OL tools. Barriers -internet problems (93%), poor internet connectivity (84%), lack of OL skills (50%), and technical problems (35%)	1
Puljak et al. (2020) [54]	OL; UG health sciences students from 9 Croatia universities	Online questionnaire	Traditional learning	(*N* = 2520) Cross-sectional study	48.5% agreed that they were equally motivated in OL compared to f2f, 43.4% felt connected with each other. Most students prefer to combine f2f and OL in the future	1
Rajab et al. (2020) [55]	OL; UG medical students	Online questionnaire	Traditional learning	(*N* = 139) Cross-sectional study	59% had communication problems, 57.5% had problems in assessment, 35.5% stated learning curve, 48% reported anxiety and stress, 17% were technophobia,	1
Sandhaus et al. (2020) [56]	OL; UG medical students from Adelson School of Medicine	Online questionnaire and telephone interview	Traditional learning	(*N* = 70) Cross-sectional study	quality of teaching was rated high at 85.7%, training and technical assistance were rated high at 87.2%. Barriers -61.5% reported having technical difficulties during OL	1
Sawarkar et al. (2020) [28]	OL; UG medical students from Bachelor of Ayurvedic Medicine and Surgery	Online questionnaire	Blended learning	(*N* = 189) Cross-sectional study	58.9% supported OL, while 33.9% were neutral. Few challenges include misconceptions, language barriers, and problem with terminologies. 54% agreed that OL engagement, increase motivation, and build their interest. However, only 37.6% supported OL	1
Schoenfeld-Tacher & Dorman (2021) [31]	OL; UG veterinary students from North Carolina State University	Online questionnaire	Traditional learning	(*N* = 103) Cross-sectional study	Students (52.6%) agreed that the OL performs almost the same with f2f, 15.8% thought OL was better. Benefits, include increased motivation and flexibility of time and place. Challenges include internet problem and lack of instructor-student interaction	2
Shahrvini et al. (2021) [57]	OL; UG medical students from the University of California San Diego	Online questionnaire	Traditional learning	(*N* = 268) Cross-sectional study	64.4% stated OL gave flexibility, 18% felt OL may cause digital fatigue, inability to focus and disengagement, 50.8% felt problems in obtaining clinical skills, and 16.7% reported feeling of loneliness	1
Sindiani et al. (2020) [58]	OL; UG medical student from Jordan university	Online questionnaire	Traditional learning	(*N* = 3700) Cross-sectional study	48.7% felt less interaction with the instructors. Benefits include saving money and energy in terms of transportation (48.7%) and less social contact thus reduced virus spreading (58.3%). Barriers include no interaction with lecturers (45.6%), technical problems (57.7%), no clinical practices (43.9%) and distraction at home (36.4%)	1
Suliman et al. (2021) [20]	OL; UG nursing students from 2 universities at Jordan	Online questionnaire	Traditional learning	(*N* = 18) Qualitative study	Students expressed worries. More time required to adapt to OL. Distraction at home was a major problem, financial burdens, lack of interaction and lack of clinical skills were mentioned. Benefits include watching the recorded lecture repeatedly, spending more time with their families, saving time, and feeling more relaxed	1
Tigaa & Sonawane (2020) [59]	OL; UG health sciences students at St. Cloud USA and College in Dhule in India	Online questionnaire	Traditional learning	(*N* = 150) Cross-sectional study	49% students from Dhule and 63% students from St. Cloud had problems with internet connectivity. 15% from Dhule and 57% from St. Cloud did not have enough electric supply to continue OL	1
Tuma et al. (2021) [60]	OL; UG medical students from Wasit University College of Medicine in Iraq	Online questionnaire	Traditional learning	(*N* = 636) Cross-sectional study	67% felt OL was difficult. 27% reported that OL met their expectations, while 67% reported not being interested and fatigued while participating in OL. Barrier- poor internet connection, unfamiliar with the OL platform, and audio-visual media quality	1
Wang et al. (2021) [61]	OL; UG dental students from 42 dental universities in mainland China	Online questionnaire	Traditional learning	(*N* = 8740) Cross-sectional study	66% agreed on increased interaction with lecturers, 88% agreed on the OL homework, 92% agreed on the OL material and 92% agreed on the effective time management. Barriers include network instability (62%), platform instability (33%), lack of learning motivation (72%), insufficient online learning ability (31%), lack of leaners-instructors interaction (59%) and others (8%)	1
Yoo et al. (2021) [62]	OL; UG medical students from Korea University College of Medicine	Online questionnaire	Traditional learning	*N* = 108 (2020) *N* = 104 (2019) Cross-sectional study	Most (89.5%) agreed that OL saved more time. 75% used that free time for self-study. Students (76.3%) repeatedly watched the recorded video. 60.5% stated that they had more available time. Barriers include internet problem (61.9%), and communication problems (50%)	1

*N* Number of students, *OL* Online Learning, *UG* Undergraduate, *f2f* face-to-face

Table 3 displays two different aspects of perceptions that students reported on online learning – positive or negative. Generally, there were more negative perceptions on online learning reported by students than positive ones. Most studies stated that internet problems (16 studies) as well as low interaction and poor communication (16 studies) contributed to the negative perceptions. In addition, seven studies reported both problems at the same time: poor internet connection as well as poor interaction and communication [19, 26, 31, 34, 45, 51, 61, 62]. This might suggest that good internet connection may facilitate good interaction and communication. Furthermore, some studies (11) stated that when students undergo online learning, that they were concerned about not being able to practice their clinical abilities. Besides that, financial difficulties might also present a major obstacle for online learning. Technological issues such as students’ and/or teachers’ inexperience with internet applications, inabilities to solve technological issues, and technophobia were also mentioned.

**Table 3:** **S**ummary of student perception of online learning based on positive and negative perception

Perception	Type of student perception	Number of studies	Study
**Positive Perception**	Flexibility	12	Al-Balas et al. (2020); Anwar et al. (2021); Bączek et al. (2021); Coffey et al. (2020); Dost et al. (2020); Gupta et al. (2021); Khalil et al. (2020); Schoenfeld-Tacher & Dorman (2021); Shahrvini et al. (2021); Suliman et al. (2021) Wang et al., (2020); Yoo et al. (2021); [19, 20, 31, 33, 37, 38, 40, 42, 45, 57, 62, 63]
	Motivated and increase engagement	7	Anwar et al. (2021); Coffey et al. (2020); Ibrahim et al. (2021); Khan et al. (2021); Puljak et al. (2020); Sawarkar et al. (2020); Schoenfeld-Tacher & Dorman (2021) [21, 28, 31, 37, 40, 46, 54]
	Save time	6	Anwar et al. (2021); Dost et al. (2020); Guiter et al. (2021); Gupta et al. (2021); Ibrahim et al. (2021); Yoo et al. (2021) [37, 42, 44–46, 62]
	High interaction between instructors and students	6	Al- Balas et al. (2020); Ibrahim et al. (2021); Martinez et al. (2020); Olum et al. (2020); Puljak et al. (2020); Wang et al. (2021) [27, 30, 33, 46, 54, 61]
	Help in clinical practices	4	De Ponti et al. (2020); Chandrasinghe et al. (2020); Co et al. (2021); Jiménez-Rodríguez & Arrogante (2020) [26, 32, 39, 41]
	Save cost	3	Co et al. (2021); Dost et al. (2020); Sindiani et al. (2020) [26, 58, 64]
	Watch and play recorded video at any time and place	3	Kim et al. (2020); Suliman et al. (2021); Yoo et al. (2021) [20, 48, 62]
	Easy to communicate	2	Guiter et al. (2021); Muflih et al. (2021) [44, 53]
	More time to study	2	Amir et al. (2020); Bączek et al. (2021) [36, 38]
	High understanding	1	Merson et al. (2020) [52]
**Negative Perception**	Internet problem	16	Al- Balas et al. (2020); Alqurshi, (2020); Chandrasinghe et al., 2020); Co et al. (2021); Dost et al., (2020); Guiter et al. (2021); Gupta et al. (2021); Ibrahim et al. (2021); Khalil et al. (2020); Menon et al. (2021); Olum et al. (2020); Schoenfeld-Tacher & Dorman et al. (2021); Tigaa & Sonawane (2020); Tuma et al. (2021); Wang et al., (2021); Yoo et al., 2021) [19, 27, 31, 33, 34, 39, 42, 44–46, 51, 59–62]
	Hard to communicate low interaction	16	Anwar et al. (2021); Alqurshi (2020); Bączek et al. (2021); Co et al. (2021); Gupta et al. (2021); Khalil et al. (2020); Khan et al. (2021); Kumar et al. (2020); Langegård et al. (2021); Menon et al. (2021); Rajab et al. (2020); Schoenfeld-Tacher & Dorman et al. (2021); Sindiani et al. (2020); Suliman et al. (2021) Wang et al. (2021); Yoo et al. (2021) [19–22, 26, 31, 34, 37, 38, 45, 49, 51, 55, 58, 61, 62]
	Cannot apply clinical skills	11	Al- Balas et al. (2020); Alsoufi et al. (2020); Alqurshi (2020); Coffey et al. (2020); Gupta et al. (2021); Ibrahim et al. (2021); Khan et al. (2021); Kumar et al. (2020); Mahdy (2020); Shahrvini et al. (2021); Sindiani et al. (2020) [21, 33–35, 40, 45, 46, 49, 50, 57, 58]
	Lack technology experience	6	De Ponti et al. (2020); Ibrahim et al. (2021); Muflih et al. (2021); Olum et al. (2020); Sandhaus et al. (2020); Wang et al., (2021) [27, 41, 46, 53, 56, 61]
	Technological problems	6	Bączek et al. (2021); Langegård et al. (2021); Martinez et al. (2020); Olum et al. (2020); Sindiani et al. (2020); Suliman et al. (2021) [20, 22, 27, 30, 38, 58]
	Related to stress and anxiety	5	Coffey et al. (2020); Dost et al. (2020); Jaap et al. (2021); Rajab et al. (2020); Suliman et al. (2021) [20, 29, 40, 42, 55]
	Technophobia	3	Rajab et al. (2020); Shahrvini et al. (2021); Tuma et al. (2021) [55, 57, 60]
	Family distraction	3	Dost et al. (2020); Sindiani et al. (2020); Suliman et al. (2021) [20, 42, 58]
	Financial problems	3	Amir et al. (2020); Suliman et al. (2021); Tigaa & Sonawane (2020) [20, 36, 59]
	Time management	3	Amir et al. (2020); Coffey et al. (2020); Langegård et al. (2021) [22, 36, 40]
	Lack motivation	2	Langegård et al. (2021); Wang et al. (2021) [22, 61]
	Behavioral challenges	2	Khalil et al. (2020); Suliman et al. (2021) [19, 20]
	Lack space at home	2	Dost et al. (2020); Jaap et al. (2021) [29, 42]
	Hard to focus (longer period)	2	Alqurshi (2020); Amir et al. (2020) [34, 36]
	More time required to prepare	2	Elsalem et al. (2021); Suliman et al., (2021) [20, 43]
	Low teaching quality	1	Al- Balas et al. (2020) [33]
	Different time zone	1	Co et al. (2021) [26]
	Lack of networking	1	Muflih et al. (2021) [53]

On a similar note, students' comprehension of their subject matter may also be hampered by psychological issues such as stress and worry, lack of motivation, and difficulties in maintaining focus during classes. The disadvantages resulting from these challenges were low teaching quality, increased behavioral challenges, lots of family distractions, lack of studying spaces at home, lack of networking, difficulties in maintaining focus during long lectures, poor time-management, and increased class preparation time due to students living in different time zones than their universities and professors. On the other hand, some studies have also recorded students mentioning several advantages to online learning, which include: higher flexibility in terms of their daily schedule, less time spent on traveling to classes, lower associated costs, easier to communicate with teachers and peers, increased engagements due to higher motivation to attend classes, more time for self-study, lessons learnt online helped in clinical practices, students are able to watch and play recorded lectures at any time and place, higher interactions between students and instructors, as well as higher understanding of course content when delivered online.

### Student satisfaction

There are 36 studies that examined students’ satisfactions from online learning (Table 4). These studies were conducted in Asia (23 studies), Europe (10 studies), Africa (2 studies), and America (1 study). In 24 out of the 36 studies (66.7%), significant results were found to favor online learning, while the remaining 12 (33.3%) were against it. The results were categorized into 3 levels of satisfaction which include dissatisfied, moderately satisfied, and highly satisfied. If the satisfaction of the students mentioned by the authors is under 40%, the study falls under the “dissatisfied” category. Any studies reporting scores between 40 to 70% were considered as “moderately satisfied”, while those that are more than 70% were considered as “highly satisfied”.

**Table 4:** Summary of the included studies for student satisfaction

Study	Intervention (what used, who involved)	Assessment tool	Comparison	Study design	Findings	Kirkpatrick
Al-Balas et al. (2020) [33]	OL; UG medical students from all medical universities in Jordan	Online questionnaire	Traditional learning	(*N* = 652) Cross-sectional study	26.77% satisfied with OL, 44.42% were not satisfied while 28.81% were neutral	1
Alsoufi et al. (2020) [35]	OL; UG medical students from all medical universities in Libya	Online questionnaire	Traditional learning	(*N* = 3348) Cross-sectional study	64.7% disagreed with OL in Libya	1
Al-Taweel et al. (2021) [65]	OL; UG dental students from University of Baghdad, University of Sulaimani, and Dijlah University College	Online questionnaire	Traditional learning	(*N* = 832) Cross-sectional study	About 79% of the participants were not satisfied with TB learning, while only 17% agreed that OL is better than traditional learning. Most of the students satisfied with TB learning mostly have advanced computer skills – 5th-grade students (Higher computer skills = higher satisfaction) – Odds Ratio: 3.031, 2.876, 3.644	1
Amir et al. (2020) [36]	OL; UG dental student from Dentistry faculty in Indonesia	Online questionnaire	Traditional learning	(*N* = 301) Cross-sectional study	44.2% of the students preferred OL to f2f learning	1
Bączek et al. (2021) [38]	OL; UG medical students from Polish	Online questionnaire	Traditional learning	*N* = 804 Cross-sectional study	o OL was not that effective compared to f2f in terms of increasing knowledge (*P* < .001) and social competencies (*P* < .001). However, 73% enjoyed OL, while 27% did not enjoy OL	1
Bolatov et al. (2021) [66]	OL; UG medical students from Astana Medical University	Online questionnaire	Traditional learning	(*N* = 619: TL), (*N* = 798: OL) Cross-sectional study	50.4% were satisfied with their academic performance during TL, while 71.6% of the students satisfied with their academic performance during OL	1
Co & Chu (2020) [67]	OL; UG medical students from the University of Hong Kong	Online	Traditional learning	(*N* = 30) Cross-sectional study	90% felt that web-based surgical skill learning (WSSL) implementation was easy to understand, and most of them did not have technical problems	1
De Ponti et al. (2020) [41]	OL; UG medical students from the University of Insubria	Online questionnaire	Traditional training	(*N* = 115) Cross-sectional study	90% gave positive feedback. 93% felt that OL was impressive	1
Dutta et al. (2021) [68]	OL; UG medical and nursing students across India	Online questionnaire	Traditional learning	(*N* = 1068) Cross-sectional study	37.76% were very satisfied with OL, while 42% were not satisfied with OL	1
Elsalem et al. (2021) [43]	OL; UG medical sciences students from Jordan University of Sciences and Technology	Online questionnaire	Traditional learning	(*N* = 1019) Cross-sectional study	Most students (91%) reported that online exam is more stressful compared to f2f while 23.55% were against the statement	1
Elzainy et al. (2020) [69]	OL; UG medical students from College of Medicine Qassim University	Online questionnaire	Traditional learning	(*N* = 249) Cross-sectional study	58.82% of students showed satisfaction with OL in virtual classrooms, virtual workshops, and online assessments	2
Fischbeck et al. (2020) [70]	OL; UG medical psychology and medical sociology from university in Mainz	Online questionnaire	Traditional learning	(*N* = 203) Cross-sectional study	Overall, most of the students agreed that all the exercise given online was very helpful while only 24% did not agree that OL can replace f2f	3
Gupta et al. (2021) [45]	OL; UG medical students from the Delhi-NCR region	Online questionnaire	Traditional learning	(*N* = 248) Cross-sectional study	35.4% of students preferred OL while 43.1% are more into f2f., 21.4% remained neutral	1
Higgins et al. (2020) [71]	OL (online problem-based learning (PBL) sessions); UG medical students from the College of Medicine at Qassim Universityf	Online questionnaire	Traditional learning	(*N* = 674) Cross-sectional study	67.30% were satisfied with OL and 64% agreed that live streaming session via Blackboard (OL platform) was efficient	1
Jiménez-Rodríguez et al. (2020) [47]	OL; UG nursing students from the University of Spain	Online questionnaire	Traditional learning	(*N* = 48) Cross-sectional study	97.6% agreed that they had learned the simulation's mistakes, 97.6% agreed that the simulation was related to the theory. Overall satisfaction showed that 95.8% felt that the simulation video was helpful	3
Jiménez-Rodríguez & Arrogante (2020) [32]	OL; UG nursing students from University in Almeria—Spain	Online questionnaire	Traditional practical	(*N* = 93) Cross-sectional study	97.8% were satisfied with the OL consultations	3
Perron et al. (2020) [64]	OL; UG medical students from Geneva Faculty of Medicine	Online questionnaire	Traditional seminars	(*N* = 149) Cross-sectional study	Students were very satisfied with the online seminars held by the faculty. eventhough students preferred f2f activity, but 60% considered OL to be used, during pandemic	1
Kaliyadan et al. (2020) [72]	OL; UG medical students from the Dermatology department	Online questionnaire	Traditional learning	(*N* = 45) Cross-sectional study	students were satisfied with the OL since all scores were more than half – Practical skills (3.28), Technical issue (3.60), Time (3.77), Assessment (3.35). Overall content coverage (3.97). The results show a lower score for practical skills since practical usually need skills that required f2f	1
Kalleny (2020) [73]	OL; UG medical students from the Faculty of Medicine Ain Shams University	Online questionnaire	Traditional learning	(*N* = 136) Cross-sectional study	Students were satisfied with Kahoot. The overall rating score is 4.65 out of 5	1
Khalaf et al. (2020) [74]	OL; UG dental students at University of Sharjah	Online questionnaire	Traditional learning	(*N* = 65) Cross-sectional study	Students were very satisfied with the online exam. Students with online exam experience were more satisfied with OL than students who lack experience with online exam (*p* < 0.05)	1
Khan et al. (2021) [21]	OL; UG medical students from North India	Online questionnaire	Traditional learning	(*N* = 103) Mixed study	62%-80%, showed satisfaction toward OL	1
Kim et al. (2020) [48]	OL; UG medical students from Seoul National University	Online questionnaire	Traditional learning	(*N* = 456) Cross-sectional study	62.2% of the students were satisfied with OL compared to f2f learning	1
Liu et al. (2021) [75]	OL; UG medical students from Shandong First Medical University	Online questionnaire	Traditional learning	(*N* = 512) Cross-sectional study	71.3% of the Histology and Embryology students and 82.5% and Pathology students were very satisfied with OL. Only 37% want to return to traditional teaching from Pathology course while 52.1% from Histology and Embryology	1
Menon et al. (2021) [51]	OL; UG medical students from college hospital in South India	Online questionnaire	Traditional learning	(*N* = 370) Cross-sectional study	most students were satisfied with the OL (31% scored high satisfaction, 53.6% scored moderate satisfaction), while only 15.4% were not satisfied	1
Muflih et al. (2021) [53]	OL; UG health science students from Jordanian universities	Online questionnaire	Traditional learning	(*N* = 1210) Cross-sectional study	The mean score obtained for students' satisfaction towards OL was 42.94,	1
Puljak et al. (2020) [54]	OL; UG health sciences students from 9 Croatia universities	Online questionnaire	Traditional learning	(*N* = 2520) Cross-sectional study	student satisfaction towards OL is 3.7 out of 5. 39.6% agreed that OL was effective, 24.9% found OL was not effective,	1
Rajab et al. (2020) [55]	OL; UG medical students	Online questionnaire	Traditional learning	(*N* = 139) Cross-sectional study	66.9% reported a positive view on the online learning application, 27.3% reported a negative view, while 5.8% reported no response	1
Sandhaus et al. (2020) [56]	OL; UG medical students from Adelson School of Medicine	Online questionnaire and telephone interview	Traditional learning	(*N* = 70) Cross-sectional study	students (88.6%) are very satisfied with OL compared to traditional learning	1
Schlenz et al. (2020) [76]	OL; UG dental students from Justus-Liebig-University Giessen (Germany)	Online questionnaire	Traditional learning	(*N* = 299) Cross-sectional study	36.8% of student prefer f2f learning, only 5.6% stated that OL is not that useful, and mostly half of them did agree that OL is useful. Overall assessment regarding student perspective was obtained, which is (53.2%—mean) (24.9—standard deviation)	1
Sindiani et al. (2020) [58]	OL; UG medical student from Jordan university	Online questionnaire	Traditional learning	(*N* = 3700) Cross-sectional study	75% were not satisfied with the OL and did not wish to use it even in the future	1
Steehler et al. (2021) [77]	OL; UG medical students from Emory University School of Medicine	Online questionnaire	Traditional learning	(*N* = 12) Cross-sectional study	92% reported that they were very satisfied with the OL. 92% agreed that there is increased understanding from this virtual learning application	1
Suppan et al. (2021) [24]	OL; UG medical students University of Geneva Faculty of Medicine	Online questionnaire	Traditional learning	(*N* = 158) RCT	Only 40% of students are very satisfied with the OL compared to 15% of traditional didactic videos	2
Tigaa & Sonawane (2020) [59]	OL; UG health sciences students at St. Cloud USA and College in Dhule in India	Online questionnaire	Traditional learning	(*N* = 150) Cross-sectional study	24% from Dhule and 15% from St. Cloud were satisfied with OL, 36% from Dhule and 22% from St. Cloud, only partially agreed with the online learning practices	1
Wang et al. (2020) [61]	OL; UG medical students from 90 medical schools in China	Online questionnaire	Traditional learning	(*N* = 118,030) Cross-sectional study	64.97% believed that OL is not effective, and 3.75% said that OL is useless. The regression results obtained showed 68.72% were not satisfied with OL	1
Yoo et al. (2021) [62]	OL; UG medical students from Korea University College of Medicine	Online questionnaire	Traditional learning	*N* = 108 (2020) *N* = 104 (2019) Cross-sectional study	most students, (78.6%), preferred OL compared to f2f learning (21.2%)	1
Zhang et al. (2020) [78]	OL; UG medical students from Zhejiang University	Online questionnaire	Traditional learning	(*N* = 48) Cross-sectional study	most students, (54.17%) preferred f2f teaching compared to the OL. Students felt that OL could provide many advantages (mean 3.83 and SD 0.95), but still, they do not think it can replace traditional learning (mean 3.87 and SD 0.94)	1

*N* Number of students, *OL* Online Learning, *UG* Undergraduate, *f2f* face-to-face

A cross-continent comparison of the level of satisfaction was also conducted. From the 13 studies that reported higher satisfaction with the use of online learning approach, six were from Asia [26, 51, 56, 62, 74, 75], five were from Europe [32, 41, 47, 66], one from America [77], and one from Africa [73]. Meanwhile, five studies from Asia [43, 58, 61, 65, 68] and one study from Africa [35] revealed that students were not satisfied with online learning. The remaining twelve studies from Asia and five studies from Europe [24, 54, 64, 70, 76] suggested that students were moderately satisfied.

A comparison of Asian and non-Asian countries revealed that most studies conducted in the former reported that more than half students were moderately satisfied (52.2%) while only around one-fifth of them were dissatisfied (21.7%) with online learning. On the other hand, students in Western countries are more likely to show higher satisfaction with online classes (53.8%). However, the differences were not statistically significant (*p*-value = 0.214).

### Learning outcomes

Fourteen studies reported learning outcomes that may be categorized into two types: 1) Based on academic performance during online learning (whether students’ performance increased, decreased, or not affected); and 2) Based on skills obtained during the online learning approach (clinical or communication skills). A summary of the included studies for student learning outcomes is presented in Table 5. According to the results obtained from data analysis, seven studies examined students’ academic performance, while the remaining seven examined the skills obtained during online learning (Table 6). Five studies from the former category reported increases in academic performance attributed to online learning while one study reported a decrease. On the other hand, one study reported that online learning did not affect students’ academic performance. In the aspect of gained skills (the latter of the two categories), two studies found that online learning helped students in enhancing their communication skills while five others found that it helped in improving students’ clinical skills.

**Table 5:** Summary of the included studies for learning outcome

Study	Intervention (what used, who involved)	Assessment tool	Comparison	Study design	Findings	Kirkpatrick
Afonso et al. (2020) [79]	OL (Virtual Respiratory Case-Based Module); UG medical students	Online questionnaire	Traditional learning	(*N* = 122) Cross-sectional study	For OL method, 93% improved their telemedicine communication, 84% in the interpretation of physical exams, 95% in the development of differential diagnosis, and 93% in seeing the correlation between clinical and basic science content	3
Amer & Nemenqani (2020) [80]	OL; UG medical students from College of Medicine, Taif University	Online questionnaire	Traditional learning	(*N* = 166) Cross-sectional study	72% preferred OL, 61.5% believed that OL was very helpful, especially during pandemic situations, and 72% agreed that OL could help them imagine the structure	3
Atli et al. (2021) [25]	OL; UG medical students at University Hospitals Cleveland Medical Center in Cleveland, Ohio, USA	Online questionnaire	Traditional learning	(*N* = 12) Prospective study	Students (100%) reported increased confidence in analyzing patients' cases and interpreting lab results. 50% agreed that VR helps them understand neuroanatomy and neurosurgery, and 66% agreed that VR helps them retain surgical skills throughout this course	3
Co et al. (2021) [26]	OL; UG medical students in Hong Kong university	Online questionnaire	Traditional learning	(*N* = 62) Case–control study	Students can do a proper surgical knot via instrumental tie. The mean score obtained for the control groups was 4.8 out of 5, while for case groups were 4.7 out of 5	3
Elzainy et al. (2020) [69]	OL; UG medical students from College of Medicine Qassim University	Online questionnaire	Traditional learning	(*N* = 249) Cross-sectional study	A significant increase of PBL marks during online sessions compared to f2f learning	2
Fischbeck et al. (2020) [64]	OL; UG medical psychology and medical sociology from university in Mainz	Online questionnaire	Traditional learning	(*N* = 203) Cross-sectional study	91% agreed that OL help them become more familiar with medical conservation practice. 76% agreed that the OL exercise helped develop communication skills	3
Higgins et al. (2020) [71]	Online training (RiTe module); UG students Bachelor of Science Honors from Northwest England Region University	Online questionnaire	Traditional training	(*N* = 44) Cross-sectional study	Students felt that the application of RiTe was very positive, They strongly agreed that RiTe was very relevant to their practice and agreed that they could also learn to master their skills from the module	2
Jaap et al. (2021) [29]	OL; UG medical students from UK medical school	Online questionnaire	Traditional learning	(*N* = 447) Cross-sectional study	Year 4 students performed better during the online exam (76.53%) compared to f2f exam (72.81%), while for Year 5students the online exam results (76.02%) were almost the same as to f2f exam (77.25%)	2
Jiménez-Rodríguez et al. (2020) [47]	OL; UG nursing students from the University of Spain	Online questionnaire	Traditional learning	(*N* = 48) Cross-sectional study	62.5% agreed that the OL simulation has improved their clinical skills	3
Kim et al. (2020) [48]	OL; UG medical students from Seoul National University	Online questionnaire	Traditional learning	(*N* = 456) Cross-sectional study	No significant differences in student academic achievement	1
Mahdy (2020) [50]	OL; UG veterinary students from 92 different countries	Online questionnaire	Traditional learning	(*N* = 1392) Cross-sectional study	96.7% agreed that OL during pandemic affected their academic performance	1
Martinez et al. (2020) [30]	OL; UG medical students from Florida Atlantic University	Online questionnaire	Traditional learning	(*N* = 47) Cross-sectional study	Students’ performance during virtual OSCE (93%) was similar to f2f OSCE (93.5%)	2
Schoenfeld-Tacher & Dorman (2021) [31]	OL; UG veterinary students from North Carolina State University	Online questionnaire	Traditional learning	(*N* = 103) Cross-sectional study	Students scored higher in diagnostic toxicology quiz online (2020) compared to f2f learning (2019)	2
Suppan et al. (2021) [24]	OL; UG medical students University of Geneva Faculty of Medicine	Online questionnaire	Traditional learning	(*N* = 158) RCT	Students who participated in OL performed very well compared to f2f	2

*N* Number of students, *OL* Online Learning, *UG* Undergraduate, *f2f* face-to-face, *RCT* Randomized Clinical Trial

**Table 6:** Summary of the different type of learning outcome

Type of learning outcome	Result	Number of studies	Study
Based on academic performance	Increase	5	Elzainy et al. (2020); Jaap et al. (2021); Kim et al. (2020); Schoenfeld-Tacher & Dorman et al. (2021); Suppan et al. (2021) [24, 29, 31, 48, 69]
	Decrease	1	Mahdy (2020) [50]
	Not effected	1	Martinez et al. (2020) [30]
Based on the type of skills obtained	Improve communication skill	2	Afonso et al. (2020); Fischbeck et al. (2020) [70, 79]
	Increase clinical skills	5	Higgins et al. (2020); Jiménez-Rodríguez et al. (2020); Amer & Nemenqani. (2020); Co et al. (2021); Atli et al. (2021) [25, 26, 47, 71, 80]

### Kirkpatrick evaluation

Overall, Kirkpatrick evaluation in Table 7 shows that fifty-one studies are at level 1, five are at level 2 [24, 29–31, 69], and eight are at level 3 [25, 32, 47, 70, 71, 79, 80]**.**

**Table 7:** Summary for Kirkpatrick evaluation for all included studies

Kirkpatrick evaluation	Number of studies	Remarks
Level 1 (Reaction)	51	Based on how participants respond to learning
Level 2 (Learning)	5	Based on how much participant learnt from the learning
Level 3 (Behavior)	8	Based on how participants applied what they learn

### Quality assessment

A quality assessment was carried out using the Alberta Heritage Foundation for Medical Research (AHFMR). The results for quality assessment of the included studies were summarized in Tables 8 and 9. Most quantitative studies (62 studies) lack the following three items: 5 (“If the random allocation was possible”); 6 (“If blinding of investigators was possible”); and 7 (“If blinding of subjects was possible”). Only two studies display a percentage lower than 50% [31, 44] while the remaining 61 registered a score of more than 50% each. Two studies were qualitative in nature [19, 20] and the percentage scored by the two is more than 50% each.

**Table 8:** Summary of quality assessment for quantitative included studies

Study	Item 1 (No N/A)	Item 2 (No N/A)	Item 3	Item 4 (No N/A)	Item 5	Item 6	Item 7	Item 8	Item 9	Item 10	Item 11	Item 12	Item 13 (No N/A)	Item 14 (No N/A)	Per 22	Per 28	< 50%	> 50%
Afonso et al. (2020) [79]	2	2	2	2	N/A	N/A	N/A	1	1	0	0	0	1	2	13			59.09
Al-Balas et al. (2020) [33]	2	2	2	2	N/A	N/A	N/A	2	2	1	0	0	2	2	17			77.27
Alkhowailed et al. (2020) [81]	2	2	2	2	N/A	N/A	N/A	2	2	0	0	0	2	2	16			72.73
Alsoufi et al. (2020) [35]	2	2	2	2	N/A	N/A	N/A	2	2	2	0	0	2	2	18			81.82
Al-Taweel et al. (2021) [65]	2	2	2	2	N/A	N/A	N/A	2	2	1	0	0	2	2	17			77.27
Alqurshi (2020) [34]	2	2	2	2	N/A	N/A	N/A	2	2	0	0	0	2	2	16			72.73
Amir et al. (2020) [36]	2	2	2	2	N/A	N/A	N/A	2	2	2	0	0	2	2	18			81.82
Amer & Nemenqani (2020) [80]	2	1	2	2	N/A	N/A	N/A	1	1	0	0	0	1	2	12			54.55
Anwar et al. (2021) [37]	2	2	2	2	N/A	N/A	N/A	2	1	0	0	0	2	2	15			68.18
Atli et al. (2021)	2	2	2	2	N/A	N/A	N/A	2	1	1	0	0	2	1	16			72.73
Bączek et al. (2021) [38]	1	2	2	2	N/A	N/A	N/A	2	2	2	2	0	2	2	19			86.36
Bolatov et al. (2021) [66]	2	2	2	2	N/A	N/A	N/A	2	2	1	0	0	2	2	17			77.27
Chandrasinghe et al. (2020) [39]	1	2	2	2	N/A	N/A	N/A	2	2	0	0	0	2	2	15			68.18
Caton et al. (2021) [23]	1	2	2	2	N/A	N/A	N/A	1	1	1	1	0	1	2	14			63.64
Co & Chu (2020) [67]	1	2	2	2	N/A	N/A	N/A	1	1	0	0	0	1	2	12			54.55
Co et al. (2021) [26]	2	2	2	2	N/A	N/A	N/A	2	1	1	0	1	2	1	16			72.73
Coffey et al. (2020) [40]	2	2	2	2	N/A	N/A	N/A	2	1	0	0	0	2	2	15			68.18
De Ponti et al. (2020) [41]	2	2	2	2	N/A	N/A	N/A	2	1	2	0	0	2	2	17			77.27
Dost et al. (2020) [42]	2	2	2	2	N/A	N/A	N/A	2	2	0	0	0	2	2	16			72.73
Dutta et al. (2021) [68]	1	2	2	2	N/A	N/A	N/A	2	2	1	0	0	2	2	16			72.73
Elsalem et al. (2021) [43]	2	2	2	2	N/A	N/A	N/A	2	2	1	0	0	2	1	16			72.73
Elsalem et al. (2021) [43]	2	2	2	2	N/A	N/A	N/A	2	2	2	0	0	2	2	18			81.82
Elzainy et al. (2020) [69]	2	2	2	2	N/A	N/A	N/A	1	1	1	0	0	1	1	13			59.09
Fischbeck et al. (2020) [70]	1	2	2	2	N/A	N/A	N/A	2	1	0	0	0	2	2	14			63.64
Guiter et al. (2021) [44]	1	1	1	1	N/A	N/A	N/A	1	1	0	0	0	1	1	8		36.36	
Gupta et al. (2021) [45]	2	2	2	2	N/A	N/A	N/A	2	1	2	0	0	2	2	17			77.27
Higgins et al. (2021) [71]	2	2	2	2	N/A	N/A	N/A	1	1	0	0	0	1	2	13			59.09
Ibrahim et al. (2021) [46]	2	2	2	2	N/A	N/A	N/A	2	2	2	0	2	2	2	20			90.91
Jaap et al. (2021) [29]	1	1	2	1	N/A	N/A	N/A	1	2	2	2	0	2	1	15			68.18
Jiménez-Rodríguez & Arrogante (2020) [32]	2	2	2	2	N/A	N/A	N/A	2	1	0	0	0	2	2	15			68.18
Jiménez-Rodríguez et al. (2020) [47]	2	2	2	2	N/A	N/A	N/A	2	1	0	0	0	2	2	15			68.18
Perron et al. (2020) [64]	2	2	2	2	N/A	N/A	N/A	1	1	0	0	0	1	2	13			59.09
Kaliyadan et al. (2020) [72]	2	2	2	2	N/A	N/A	N/A	1	1	0	0	0	1	2	13			59.09
Kalleny (2020) [73]	2	2	2	2	N/A	N/A	N/A	1	1	0	0	0	1	2	13			59.09
Khalaf et al. (2020) [74]	2	2	2	2	N/A	N/A	N/A	1	1	1	0	0	1	2	14			63.64
Khan et al. (2021) [21]	2	2	2	2	N/A	N/A	N/A	2	1	1	0	0	1	2	15			68.18
Kim et al. (2020) [48]	2	2	2	2	N/A	N/A	N/A	2	2	1	0	0	2	2	17			77.27
Kumar et al. (2020) [49]	2	1	1	2	N/A	N/A	N/A	1	1	0	0	0	2	1	11			50.00
Langegård et al. (2021) [22]	2	2	2	2	N/A	N/A	N/A	2	2	0	0	0	2	2	16			72.73
Liu et al. 2021) [75]	2	2	2	2	N/A	N/A	N/A	2	2	2	0	0	2	2	18			81.82
Mahdy (2020) [50]	2	2	2	2	N/A	N/A	N/A	1	2	0	0	0	1	2	14			63.64
Martinez et al. (2020) [30]	1	2	2	2	N/A	N/A	N/A	1	1	0	0	0	1	2	12			54.55
Menon et al. (2021) [51]	2	1	2	2	N/A	N/A	N/A	1	2	1	0	0	1	2	14			63.64
Merson et al. (2020) [52]	2	2	2	2	N/A	N/A	N/A	1	1	2	0	0	1	2	15			68.18
Muflih et al. (2021) [53]	2	2	2	2	N/A	N/A	N/A	2	2	2	2	0	2	2	20			90.91
Olum et al. (2020) [23]	2	2	2	2	N/A	N/A	N/A	1	1	2	0	0	1	2	15			68.18
Puljak et al. (2020) [54]	2	2	2	2	N/A	N/A	N/A	2	2	0	0	0	2	2	16			72.73
Rajab et al. (2020) [55]	2	2	2	2	N/A	N/A	N/A	2	1	1	0	0	2	2	16			72.73
Sandhaus et al. (2020) [56]	2	2	2	2	N/A	N/A	N/A	2	1	1	0	0	2	2	16			72.73
Sawarkar et al. (2020) [28]	2	2	2	2	N/A	N/A	N/A	2	1	0	0	0	2	2	15			68.18
Schlenz et al. (2020) [76]	2	2	2	2	N/A	N/A	N/A	2	1	1	2	0	2	2	18			81.82
Schoenfeld-Tacher & Dorman (2021) [31]	1	1	1	2	N/A	N/A	N/A	1	1	1	0	0	1	0	9		40.91	
Shahrvini et al. (2021) [57]	2	2	2	2	N/A	N/A	N/A	2	1	1	0	0	2	2	16			72.73
Steehler et al. (2021) [77]	2	2	2	2	N/A	N/A	N/A	1	1	0	0	0	1	2	13			59.09
Suppan et al. (2021) [24]	2	2	2	2	2	0	0	2	1	2	0	2	2	1		21		75.00
Tigaa & Sonawane (2020) [59]	2	2	2	2	N/A	N/A	N/A	2	1	0	0	0	2	2	15			68.18
Tuma et al. (2021) [60]	2	2	2	2	N/A	N/A	N/A	2	2	0	0	0	2	2	16			72.73
Sindiani et al. (2020) [58]	2	2	2	2	N/A	N/A	N/A	2	2	1	0	0	2	1	16			72.73
Wang et al. (2020) [63]	2	2	2	2	N/A	N/A	N/A	2	2	2	0	0	2	2	18			81.82
Wang et al. (2021) [61]	2	2	2	2	N/A	N/A	N/A	2	2	0	0	0	2	2	16			72.73
Yoo et al. (2021) [62]	2	2	1	2	N/A	N/A	N/A	2	1	1	0	0	2	0	13			59.09
Zhang et al. (2020) [78]	2	2	2	2	N/A	N/A	N/A	2	1	1	2	0	1	1	16			72.73

Yes = 2, Partial = 1, No = 0, N/A = non-applicable

Q1 = Question or objective sufficiently described Q2 = Design evident and appropriate to answer study question Q3 = Method of subject selection or source of information is described and appropriate Q4 = Subject characteristics or input variables sufficiently described Q5 = If the random allocation was possible, is it described? Q6 = If blinding of investigators was possible, is it reported? Q7 = If blinding of subjects was possible, is it reported? Q8 = Outcome and exposure measure well defined and robust with bias misclassification reported Q9 = Sample size appropriate Q10 = Analysis described and appropriate Q11 = Estimate of variance is reported Q12 = Controlled for confounding Q13 = Result reported in sufficient detail Q14 = Result support the conclusions

**Table 9:** Summary of quality assessment for qualitative studies

Reference	Item 1	Item 2	Item 3	Item 4	Item 5	Item 6	Item 7	Item 8	Item 9	Item 10	Per 20	> 50%
Khalil et al. (2020) [19]	2	2	2	2	2	2	2	2	2	2	20	100
Suliman et al. (2021) [20]	2	2	2	2	2	2	2	1	2	2	19	95

Note: Yes = 2, Partial = 1, No = 0

Q1 = Question or objective sufficiently described Q2 = Design evident and appropriate to answer study question Q3 = Context of the study clear Q4 = Connection to a theoretical framework/wider body of knowledge? Q5 = Sampling strategies described, relevant and justified? Q6 = Data collection method clearly describe and systematic? Q7 = Data analysis method clearly describe and systematic? Q8 = Use verification procedure to establish credibility? Q9 = Conclusions supported by results Q10 = Relativity of the account?

## Discussion

Time spent, content, and pedagogy during online learning can lead to noticeable differences in students' learning outcomes [33, 55, 82]. Nonetheless, there is still no conclusive evidence that online learning is preferable as a medium for delivering lessons [83]. Students’ level of satisfaction with online learning can be influenced by their general perceptions of such delivery method [84]. Almost 50% of the studies reviewed stated that students are moderately satisfied, 37% reported that students are highly satisfied, while only 14% asserted that the students are dissatisfied. Most students mentioned flexibility (26%) as the most important factor that contributed to their satisfaction with online learning. This is possibly because they are able to log into online applications such as Zoom or Google Meet at any time of their convenience. Some students also mentioned that they had concerns about finding time to come to campus or to meet with instructors. This is especially pronounced among students living in rural areas [85]. Students also reported that online learning has helped them to be more motivated in learning. This is the case as students’ were reported to feel more excited in learning to use new tools – such as new technologies that can be used to assist them during studying – effectively boosting their motivation [86].

Furthermore, according to six studies, online learning may allow for higher efficiency resulting in time savings. This is particularly true when certain lecturers swap traditional exams with reflective tasks like class conferences – where students must contribute by sharing their thoughts on what they understand about the lecturer's unique topic. This form of assessment has saved time for both students and lecturers as well as contributed to students’ better comprehension [87, 88]. High student-instructor interaction was also observed as online learning provides two kinds of lesson delivery tools: asynchronous and synchronous tools (such as e-mail, forums, chats, and videoconferences). These tools allow for the distribution of more content to a larger number of students and has resulted in better communication between students and instructors [89].

According to Coman et al. (2020), online learning fosters deeper understanding among students compared to traditional teaching. This improved understanding can, in turn, help students to perform better – especially in clinical practices [90]. Students also agreed that online learning helps in saving money and/or reduces costs, especially when the students do not have to incur additional expenses on transportation to commute to their physical classes [58]. Besides that, most students stated that recorded lectures during online learning are highly useful as they may re-watch the material offered at any time of their convenience. This has allowed the students to have more time for self-study and revisions [36, 62, 91].

Concerning student engagement, one study found that online learning improves this aspect significantly when compared to traditional learning methods [23]. This study utilized retrospective cohort studies to examine students’ questioning behavior in face-to-face versus online classes. According to the findings, students are more likely to ask questions during online learning than during face-to-face learning. The queries asked by students are also more complicated. The researchers concluded that this was the case as students do not need to raise their hands or speak directly to instructors to ask a question in an online learning setting. Instead, they can type their questions in the chat box and submit them anonymously. A timid student who constantly hesitates to ask questions during physical in-person class can benefit from these tools as they provide the much needed anonymity. The chat or question box will remain visible until the end of the session, which allow other students to respond to the question or participate in the discussion.

On a different note, students who were not satisfied with online learning complained that internet problems and sub-par communications between students and instructors as among the factors that contributed to their dissatisfaction. High bandwidth and a robust internet connection are required for a seamless experience during online classes. However, not all students can afford them. This has resulted in many students experiencing problems with their internet connection despite having cellular data or Wi-Fi connections at home. Sub-par communications between students and instructors may happen due to the lack of effective interactions that occur when instructors are unable to monitor their students as effectively as they could in a physical setting. In addition, instructors would not be able to meet and discuss with their students as frequently as they would like – to some student’s dismay [92]. Because students and teachers would not physically observe each other’s body language in an online setting, maintaining an effective communication has become more challenging and requires more effort than face-to-face sessions. During in-person lectures, lecturers can easily use body language and facial expressions to help students understand the content more effectively. Nevertheless, these elements are usually not present in an online setting (or not as pronounced), making communications more difficult and resulting in sub-par interactions between students and instructors [93].

According to Chan et al. (2020), experience-based learning is very important for students to gain new experiences as they participate in various activities involving patients and clinical teachers [94]. However, because of the pandemic and the associated travel restrictions, most activities can only be completed online via Zoom or Google Meet. This has directly impacted students’ performances in their clinical practices. Some students also mentioned that they are worried that missing physical clinical training during their degrees might lead them to lose their job opportunities in the future [95]. This challenge is further exacerbated as some students also lack familiarity with technology and often encounter technological issues such as incompatibility of online learning software with their computer’s operating system and the browsers they use. In addition, some cellphones can only support a limited number of applications [92]. According to Sitzmann et al. (2010), students' learning outcomes might be significantly impacted by technical difficulties leading to an increase in students’ displeasure [96].

Besides that, online learning might create anxiety and depression among students. This is especially pertinent during the Covid-19 quarantine period as university students are more likely to get stress disorders and depression due to prolonged social isolation, which can exacerbate procrastination and a sense of worthlessness [97]. Moreover, technophobia – defined as a fear of technology that stems from unfavorable encounters with it – may foster students’ hesitant attitude towards online learning [98].

As suggested Rasmitadila et al. (2020), students tend to lose attention during online learning sessions due to a variety of factors including family distractions and the lack of a conducive setting for learning [99]. Family distractions – especially for students with large immediate families and who do not have a conducive setting for learning (where students have no choice but to study in the living room while their family members are around) – can negatively impact students’ learning experience significantly. Furthermore, some students stated that they were having financial difficulties that hinder them from affording a data plan and strong Wi-Fi for online learning. In addition to that, some students asserted that time management is extremely difficult during the pandemic as they are not constantly supervised by their lecturers, effectively leading to their sub-par performances [100].

According to Gustiani (2020), online learning caused\s some students to lose motivation in their studies. This might occur due to a couple of factors including unfavorable learning environments (for example, there are parents that ask their children to do household chores during online lessons) [86]. Online learning exams have also been shown to result in behavioral changes in students – such as changing dietary behaviors, inconsistent sleeping patterns, and deterioration of physical exercise [43]. Besides that, students also complained about the length of online tests as some of them did not have enough time to answer all questions given. This could be attributed to technical issues that occurred during the online test (including lagging and/or slow laptops). Because of these issues, the students believed that more time was needed to prepare during online tests in comparison to their traditional counterparts [101]. On a similar note, in a study where the delivery of educational information via live streaming sessions by instructors required good internet bandwidth to get the best streaming quality, low teaching standards have been reported by students. [33]. According to Co et al., (2021), students reported that they were unable to collaborate with a subject matter expert throughout the online learning process due to the lack of networking [26]. Some international students also experienced difficulties due to the difference in time zones between their home countries and their universities [53].

Most studies conducted are in the field of medical education. The evaluation of the effectiveness of online learning was done based on students’ academic performance as well as the skills they obtained through the lessons. Five studies (72%) reported an increase in academic performance when compared to the traditional approach, one study (14%) reported a decrease in academic performance, while one study (14%) concluded that students are not affected by the different delivery methods. These results demonstrate that student performance can improve with the use of online learning during a pandemic. According to Gonzalez et al. (2020), during the pandemic, more students started to pass their courses and more students finished their assignments than in prior years [102]. Because of this, they suggest that the rise in students’ academic performance is related to the greater constancy in studying as the results of online learning arrangements. Finally, the improvement in students’ performance may also be attributed to the lack of distractions. Some students – particularly the low-performing ones – may be less distracted by their peers if they learn at home. This has allowed them to focus more on their studies and, as a result, improve their academic performance [103–105].

Most studies agreed that online learning could help students improve their skills such as communication and clinical skills. Two studies stated that online learning improves the former, while five studies suggested that it improves the latter. According to results obtained from Gormley et al. (2009), online learning had a positive impact on students' clinical skills [106]. Most of the students surveyed in their study agreed that the lessons on clinical capabilities that they get through online learning were on par with those obtained through traditional physical setting. Furthermore, the researchers claimed that students who exhibit characteristics related to deeper learning in clinical skills would perform better when learning online. In addition, students were also quite comfortable with the usage of internet video and photographs during clinical procedures. With regards to improvements in communication skills, Rodrigues and Vethamani (2015) found that online learning approaches may assist students in acquiring these skills [107]. Online learning can motivate students to practice their oral communication skills in a one-on-one learning environment that is critical for them to develop their self-confidence.

Based on the screened articles, the two countries that exhibit the highest number of studies not in favor of online learning applications are India [45, 59, 68] and Jordan [33, 43, 58, 108]. The biggest challenge to implement online learning as observed in India is the lack of accessibility. The overall number of internet users in India is estimated to be around 564.5 million in 2020, although the entire population in the same year was around 1.38 billion. This implies that more than half of the population lacked access to the internet during the pandemic [109]. Most Indian families face financial difficulties that hinder their children from having their own equipment such as laptops, PCs, and cell phones for online learning use. Some families with multiple children also reported having difficulties enrolling themselves in online programs and lessons, as the entire family depends on a single gadget at home that must be shared with everyone [110].

Along a similar line, the lack of electricity has also been identified as one of the hurdles of online learning, particularly for students who live in remote areas. The lack of electricity contributed to minimal internet penetration resulting in poor internet speeds [111]. According to Aljaraideh and Bataineh (2019), the lack of adequate online learning infrastructure is the most frequently reported difficulty in online learning by students in Jordan [112]. Furthermore, their study stated that the impact of the existing weak infrastructure could be compounded by the lack of proper assistance from the government and higher education's top administration.

### Kirkpatrick evaluation

Kirkpatrick Evaluation was utilized to acquire a thorough grasp of how online classes influence learning and whether there is a major difference in how students learn. 80% of the studies access the effectiveness of online learning based on Level 1 (Reaction), 8% based on level 2 (Learning), 12% based on Level 3 (Behavior), while no studies were accessed based on Level 4 (Results).

According to the Kirkpatrick evaluation, most studies reviewed were evaluated at Level 1 (Reaction), which is based on students’ “reactions” to online learning. Only a few studies were evaluated at Level 2 (Learning) and Level 3 (Behavior), while none were evaluated at Level 4 (Result). Future research should concentrate on analyzing the effectiveness of online learning at higher levels of the Kirkpatrick model – such as Level 3 (Behavior) and Level 4 (Result) – as studies performed at these levels can yield more consistent results. Furthermore, future studies should entail the usage of Randomized Clinical Trials (RCT) and qualitative research methods. This is because these study designs are more dependable (in comparison to a simple cross-sectional study design), allowing for more accurate conclusions to be drawn.

### Limitation of this study

The main limitation of this study is that it involves the review of many cross-sectional studies. Only three studies were non-cross-sectional by design – one utilized Randomized Controlled Trials (RCTs) and two others were qualitative in nature. According to Levin (2006), cross-sectional studies are not the most reliable for making causal inferences, while prejudice (Neyman bias) is more likely to emerge during the research process. RCTs have a significant benefit over other study designs that use a randomization technique [113]. Allocation bias and confounding or unknown variables can be reduced by randomly assigning individuals to the test and control groups. Compared to other study designs, RCTs can also be utilized to make causal inferences and provide the strongest empirical evidence [114]. Our study may have reached some inaccurate conclusions due to the small number of RCTs and qualitative studies screened. To summarize, in the field of education, it is not enough to just question “what works”, It is also necessary to ask “what works for whom, in what circumstances, and in connection to what” in order to reach to a sound and reliable conclusion [12].

## Conclusion

School cancellations caused by COVID-19 have caused enormous disturbances in the education sectors across various countries, significantly altering how students are educated. The efficiency of online learning was assessed in this systematic review based on a variety of parameters based on the Kirkpatrick model of evaluation. The parameters include students’ reaction and attitudes (perceptions/ satisfactions/ engagements), as well as students’ learning outcome. According to most studies, students’ overall satisfaction with online learning applications is higher vis-à-vis traditional teaching techniques. Students believed that online learning provides various advantages including greater flexibility, boosts students’ motivation, as well as offers various time and cost savings. However, most studies found that internet connectivity issues and low interaction between instructors and learners are among the most significant drawbacks of this approach. Studies that investigated learning outcomes as a major performance indicator for online learning, on the other hand, found that this learning method helps students improve their academic performance as well as clinical and communication skills.

## Data Availability

Data and materials are available from the corresponding author upon reasonable request.
